# Reconstructing lease-to-own contracts: A contemporary approach to Islamic banking standards

**DOI:** 10.1016/j.heliyon.2023.e19319

**Published:** 2023-08-19

**Authors:** Mahmoud Fayyad

**Affiliations:** Department of Private Law, College of Law, University of Sharjah, United Arab Emirates

**Keywords:** Lease-to-own contracts, *Ijārah muntahiyah bi-t-tamlīk*, Islamic banks, Islamic transactions, Palestine

## Abstract

The Palestinian Monetary Authority issued decision No. 15 of 2019 demanding Islamic banks to act according to Islamic standards set by the Accounting and Auditing Organization for Islamic Financial Institutions (AAOIFI). Among the essential financial services, Islamic banks offer *Ijārah Muntahiyah bi-t-tamlīk* contracts (lease-to-own), which share a similar purpose with financial leasing contracts. This decision places Islamic banks in a challenging predicament, caught between the modern doctrinal orientations of AAOIFI standards and the traditional orientations provided in *Al-Majjahllah* Code. The formation of *Ijārah Muntahiyah bi-t-tamlīk* contracts relies on a promise of sale issued by the bank to the tenant, contingent on the complete payment of agreed-upon installments. The first challenge arises because the general rules do not recognize the unilateral promise as a source of legal obligations, thus rendering it without legal value. The second issue stems from several contract clauses that contravene AAOIFI standards by burdening the tenant with most of the bank's obligations. Despite the importance of resolving these contradictions, no comprehensive review has been undertaken to date. This research introduces specific recommendations to ensure the consistency of these transactions with Islamic standards and relevant legislative provisions as provided in national law. By bridging this gap, the study seeks to enhance the effectiveness and compliance of lease-to-own contracts in Palestinian Islamic banks, fostering a more robust and Sharia-compliant financial system.

## 1 Introduction

The foundation of a country's economy rests on two fundamental sectors: the real sector and the monetary sector. The real sector encompasses economic activities centered around manufacturing and services. Conversely, the monetary sector is closely tied to the banking industry [1]. In Islamic economic principles, a key concept emphasizes the importance of maintaining equilibrium between these two sectors. This balance ensures that the growth of financing remains interconnected with the growth of the real sector, which is the recipient of such financing. In other words, the goal is to maintain a connection between financial growth and the actual sectors being funded [2]. The banking systems in Islamic countries, including Palestine, are structured into two main categories. The first is the conventional banking system, which operates on an interest or usury basis, where additional interest is charged on the loan amount. The second system is the Islamic banking system, which strictly adheres to Islamic principles based on the Qur'an and Hadith. This system is characterized by its unique emphasis on profit and loss sharing, setting it apart from the conventional system [1]. Islamic banking can be seen as a progressive extension of Islamic economics, particularly in the financial realm. It originated as a response to the demands of Muslim economists and banking professionals who sought to address the expectations of different stakeholders [3]. These individuals aimed to provide financial transaction services guided by moral values and principles rooted in *Sharia* law. *Sharia* law refers to a set of principles and guidelines derived from Islamic religious texts, encompassing various aspects of life for Muslims, including personal behavior, family matters, and criminal justice. Thus, Islamic banking emerged to reconcile economic practices with ethical and Sharia-compliant principles [1].

In most Muslim Countries, Islamic financial institutions have been formally established to offer alternative financial solutions following Sharia principles [4,5]. They provide diverse financial products that avoid conventional bank interest [3]. Islamic banks are financial establishments that gather, invest, and mobilize funds to promote the growth of the market economy and foster Islamic cooperation. These activities follow the principles and standards of Islamic teachings, encompassing cooperative insurance and the practice of *Zakat*, while striving to serve the best interests of their clients [6,7]. *Zakat* is an obligatory Islamic act of giving, where Muslims contribute a portion of their wealth to assist the less fortunate and those experiencing hardship. They conduct substantial financial operations in both Arab and Western countries. Based on the Islamic Finance Development Indicator (IFDI), the Islamic finance sector witnessed substantial growth in 2021, with total assets reaching nearly US$4.0 trillion, representing a remarkable 17% increase from the previous year. Additionally, the global net income reported by Islamic financial institutions experienced a three-fold increase in 2021 compared to 2020, indicating significantly improved outcomes, particularly for Islamic banks [8].

According to a recent report from *Bait Al-Mashura* for Financial Consultations, the global total value of financial assets within the domain of Islamic finance stands at $2.43 trillion, with Islamic banks and banking institutions accounting for $1.72 trillion of this sum [9,10]. The study anticipates a remarkable 56% surge in the value of their assets, projecting them to reach $3.8 trillion by 2023, supported by a steady growth rate of 15–20%. Islamic banks offer a diverse range of financing services, among which the most significant ones are *Murabaha*, *Musharaka*, and *Mudaraba*. Furthermore, they provide *Ijārah Muntahiyah bi-t-tamlīk* services which are similar-in function-to financial leasing contracts. *Murabaha* (cost-plus financing) is an Islamic financial transaction in which the seller reveals the cost and profit margin to the buyer. The buyer agrees to purchase the item at an agreed-upon markup and makes interest-free installment payments. *Musharaka* (partnership contract) is an Islamic partnership where multiple parties invest capital in a venture, sharing profits and losses based on agreed ratios, and all partners can actively manage the enterprise. *Mudaraba* (profit-sharing investment) is an Islamic financial arrangement where one party invests capital (the investor), and the other manages the investment (the entrepreneur). Profits are shared based on an agreed ratio, while the investor primarily bears any losses. In Palestine, there has been a notable escalation in the overall financial support extended by Islamic banks for these transactions, surging from $92 million in 2018 to $149.8 million in 2021. This substantial increase represents a remarkable growth rate of 63% during this specified timeframe [11,12].

It has been suggested that within the Islamic banking industry, *Ijārah* is one of the most frequently employed financial products [13]. According to Alkhan's empirical findings, this transaction is frequently used as an Islamic financial product to finance assets of higher value with longer financing tenors than other Islamic transactions. This difference in usage may be attributed to the distinct characteristics inherent in the *Mudaraba* and *Ijārah* products [14]. The rent-to-own concept was rooted in the Islamic principle of *Ijārah*, where a lessee rents an asset to acquire it. In Islamic law, *Ijārah* signifies “hiring a person's services in return for compensation.” The parties involved in this arrangement are referred to as the *Musta'jir* (lessor) and the *Ajir* (lessee) [15].

In Palestine, Islamic transactions are not governed by a specific law. Also, the Palestinian Monetary Authority (PMA) does not issue special instructions governing their transactions [16,17]. The PMA has mandated commercial banks adhere to the regulatory capital requirements stipulated by the Basel III resolutions. However, Islamic banks have been explicitly exempted from this obligation, as stated in Instruction No. 8 of 2018. On the other hand, Instruction No. 9 of 2018 imposes the responsibility on Islamic banks to adhere to capital adequacy requirements aligned with the standards issued by the Islamic Financial Services Council. This underscores the necessity for these banks to comply with the standards set forth by Islamic financial institutions. Verifying the compatibility of the standard contracts issued by Islamic banks with the Accounting and Auditing Organization for Islamic Financial Institutions (AAOIFI) standards and *Al-Majjahllah* is important. In various instances, the AAOIFI standards expressly forbid any agreement or contractual clause that contradicts its standards and the principles of Islamic jurisprudence.

To ensure the consistency of transactions offered by Islamic banks in Palestine with Sharia standards, the PMA released Decision No. 15 of 2019, granting the Supreme Shariah Supervisory Board the authority to scrutinize transactions carried out by Islamic banks. This measure aims to guarantee adherence to the standards laid out by AAOIFI. This decision aligns perfectly with Article No. 20 of Law No. 9 of 2010 governing banks in Palestine, which mandates that Islamic banks must conduct all their operations following the provisions of Islamic *Sharia*. The term *Sharia* encompasses all the beliefs and actions that *Allah* (God) has divinely legislated [18]. Since these banks are obligated to adhere to the requirements of *Sharia*, the PMA established a Sharia Supervisory Board to ensure the institution's compliance with Islamic law. The scholars on the Sharia Supervisory Board issue *Fatwas*, which are jurisprudential rulings based on the principal sources of *Sharia*, namely the *Qurʾān* and *Sunnah*. If a *Fatwa* prohibits involvement in *Riba* (interest or usury), a ruling grounded in Islamic law, then abiding by this ruling should automatically align with the intended *Maqaṣid* (objectives) of Islamic finance [18].

The AAOIFI holds a preeminent position among international non-profit organizations that provide crucial support to Islamic financial institutions. Established in 1991 and headquartered in Bahrain, it has published 100 jurisprudential standards governing Islamic financial transactions to pursue its objectives effectively. These standards are implemented in over 45 Islamic countries, including Palestine. Adherence to these standards is a fundamental aspect of any Islamic bank's dedication to conducting its business per Islamic principles, given that these standards enjoy approval from 90% of Islamic banks and financial institutions worldwide [17,19]. It is important to note that the establishment of AAOIFI was intended to supplement the International Financial Reporting Standards (IFRS). Rather, its purpose is to provide a complementary framework that addresses the disparities between Islamic and conventional finance transactions [20]. Therefore, establishing standard-setting bodies that address these gaps has become imperative. The key Islamic standard bodies include AAOIFI, the Islamic Financial Service Board (IFSB), and the International Islamic Financial Market (IIFM). Each entity shapes and promotes adherence to Islamic financial principles and practices [20]. Therefore, the current iteration of AAOIFI comprises a comprehensive collection of 103 standards as of the year 2020. The development and evaluation of these standards follow a meticulous five-stage process, commencing with the formulation of a working agenda, followed by initial studies, discussion drafts, issuance of exposure drafts, and culminating in the final version. To ensure thoroughness and expertise, this process involves the establishment of a dedicated committee composed of board members, alongside the involvement of external consultants. These measures are taken to ensure the quality, effectiveness, and adherence to Islamic financial principles in the formulation and implementation of AAOIFI standards [20,21].

Given the distinctive approaches to business transactions in Islamic and conventional financial systems, the Islamic financial sector requires complementary standards to govern its operations. These standards are derived from the *Fiqh Muamalat*, which outlines financial transaction rules [21]. Consequently, the development and acceptance of Islamic financial standards involve careful consideration of international standards to ensure compatibility and consistency. There has been a lack of national research concerning compliance with AAOIFI Standards on *Ijarah* financing. This research paper explores the level of non-compliance in the Ijarah financing practices of contemporary Islamic banks in Palestine regarding the AAOIFI Shariah standard and the general doctrinal rules governing contractual agreements. The research seeks to assist the Islamic banking industry in Palestine in bridging the gap and potentially reducing or eliminating discrepancies between the AAOIFI Shariah standards concerning Ijarah financing.

### 1.1 Research objectives

• Assess the compliance of *Ijārah Muntahiyah bi-t-tamlīk* contracts (lease-to-own) offered by Palestinian Islamic banks with the recent instructions issued by PMA and the standards set by the AAOIFI.
• Identify and analyze the challenges Islamic banks face in Palestine as they navigate the discrepancies between modern doctrinal orientations of AAOIFI standards and traditional orientations provided in *Al-Majjahllah* Code concerning *Ijārah Muntahiyah bi-t-tamlīk* contracts.
• Examine the legal implications and validity of the unilateral promise issued by Islamic banks during the formation of *Ijārah Muntahiyah bi-t-tamlīk* contracts, considering the general rules that may not recognize such promises as a source of legal obligations.
• Investigate specific clauses within the *Ijārah Muntahiyah bi-t-tamlīk* contracts that potentially violate AAOIFI standards by transferring significant obligations to the tenant. Then, propose modifications to ensure compliance with Islamic standards and relevant national legislation.

### 1.2 Research questions

• How do Palestinian Islamic banks currently structure and implement *Ijārah Muntahiyah bi-t-tamlīk* contracts in response to the recent instructions issued by the PMA and AAOIFI standards?
• What are the key challenges Islamic banks face in Palestine when attempting to reconcile modern AAOIFI standards with traditional legal orientations provided in *Al-Majjahllah* Code regarding *Ijarah Muntahiyah bi-t-tamlīk* contracts?
• What are the legal implications of the unilateral promise issued by Islamic banks during the formation of *Ijarah Muntahiyah bi-t-tamlīk* contracts, and how does this aspect affect the legal validity and enforceability of the contracts?
• Which specific clauses within the *Ijarah Muntahiyah bi-t-tamlīk* contracts may violate AAOIFI standards by burdening the tenant with excessive obligations, and what recommendations can be proposed to modify these clauses to ensure compliance with Islamic standards and national legislation?

### 1.3 Research novelty

The research contributed valuable insights to the field of Islamic banking and finance by addressing the lack of specialized legal studies on *Ijarah Muntahiyah bi-t-tamlik* contracts and providing guidance for Palestinian Islamic banks to enhance their practices following *Sharia* principles and legal requirements. In the subsequent sections, this research paper delves into an in-depth analysis of the challenges confronted by Islamic banks when implementing the AAOIFI standards in the context of lease-to-own contracts. The paper sheds light on two significant challenges that pose obstacles to the seamless application of these standards. The first challenge examines how well Islamic banking principles align with the fundamental principles of contract law provided in national law. The second challenge addresses the widespread utilization of pre-drafted contracts by Islamic banks. This practice can place a disproportionate share of responsibilities on the customer, potentially contradicting the core values of Islamic finance that prioritize fairness and balanced sharing of duties among the participants in a financial transaction. The research paper critically evaluates the impact of such standard-form contracts on the overall dynamics of lease-to-own agreements. It proposes alternative approaches that adhere more closely to the principles of Islamic banking.

### 1.4 Research limitation

As the primary objective of this research paper is to offer thorough perspectives on the alignment of *Ijārah Muntahiyah bi-t-tamlīk* contracts provided by Palestinian Islamic banks with regulatory guidelines and AAOIFI standards, along with an examination of challenges arising from doctrinal differences and legal consequences of unilateral commitments, it is essential to recognize specific constraints that could influence the extent and applicability of the outcomes:

• Evolving regulatory landscape: While the study centers on the recent directives from PMA and AAOIFI standards, it is crucial to recognize the dynamic nature of the regulatory framework. Potential post-study modifications or regulations additions might influence the research findings' precision and pertinence.
• Emphasis on qualitative methodology: The research predominantly embraces a qualitative methodology to examine challenges and disparities. While this approach yields valuable insights, it may not encompass the entire quantitative scope of challenges encountered by Islamic banks or present a comprehensive statistical portrayal.
• Applicability to different jurisdictions: The findings and suggested adjustments could be tailored to the distinct Palestinian legal and banking setting. Caution should be exercised when extending these findings to other geographical areas or nations with varying legal structures and cultural backgrounds.
• Time limitations: The exhaustive exploration of all facets related to *Ijārah Muntahiyah bi-t-tamlīk* contracts and their associated challenges might encounter constraints due to time considerations. Certain intricate elements might necessitate more extensive research beyond the boundaries of this study's scope.

Nevertheless, despite these acknowledged limitations, this research paper strives to offer significant insights into the realms of compliance, challenges, and prospective enhancements concerning *Ijārah Muntahiyah bi-t-tamlīk* contracts provided by Palestinian Islamic banks.

## 2 Literature review

Numerous scholars have varying definitions of leasing [2]. The Hanafi school, the source of *Al-Majjahllah* Code, defines it as a contract of benefits with offset. The *Maaliki* school sees it as a permissible benefit for informed individuals with offset. The *Shaafa* school defines it as a contract allowing unintended benefits of information with an offset. The *Hanbali* school characterizes it as a contract for permissible benefits known for a specific period, from a known party, described in the disclosure, or related to a known work. Researchers consider the *Hanbali* definition more accurate as it encompasses other definitions and adds necessary conditions. Leasing can be categorized into leasing described in the disclosure, leasing of objects, and leasing to individuals [2]. In conclusion, the *Ijarah Muntahiyah bi-t-tamlīk* is defined as: “It is an agreement in which an Islamic bank acquires an asset at the client's request, making a spot payment for it. Subsequently, the bank leases the asset to the client and commits, through a separate document, to transfer ownership of the asset after the client fulfills all payment obligations” [11,22]. Due to their increasing impact on economic and human development, small and medium-sized enterprises (SMEs) have gained global attention, making this contract crucial in financing them [10,11,23]. Small and medium-sized projects encounter challenges in securing funding for their fixed capital, both during their establishment and throughout the growth of their commercial operations. Unlike larger firms, these projects cannot access financial markets and raise funds by issuing shares and bonds, further complicating their financing prospects [7,16]. Moreover, they cannot fulfill their financing requirements entirely through commercial banks, as these banks often exploit their situation by imposing exorbitant interest rates and unfavorable terms [24,25]. These transactions offer ideal solutions for these firms, ensuring the equity of contractual terms by adhering to the Islamic *Sharia* principles discussed in this research segment.

### 2.1 Islamic policy in governing *Ijārah Muntahiyah bi-t-tamlīk* transactions

The *Ijarah Muntahiyah bi-t-tamlik* differs from financial leasing provided by commercial institutions. In the latter, the sale and lease provisions are applied concurrently, and ownership is transferred to the lessee once the agreed installments are fully paid, eliminating the need for a separate contract [[26], [27], [28]]. Regarding the *Ijarah Muntahiyah bi-t-tamlik* transactions, the lease contract's provisions govern the legal relationship between the parties involved for the entire lease duration. Alongside the lease contract, the two parties also enter a sales promise, wherein the owner commits to transferring the property to the lessee once all the financial installments, as agreed upon in the lease contract, are paid. Subsequently, the ownership of the leased property is transferred to the lessee under a new agreement based on this promise [[29], [30], [31], [32]]. Following the traditional *Sharia* principles, if a bank intends to act on behalf of a customer in purchasing goods from third parties, the leasing transaction should only occur after the item has been acquired by the bank. This principle aligns with the views of scholars, as elucidated by *al-Zuḥaylī*, who assert that engaging in transactions involving goods not yet owned is considered impermissible due to the presence of manipulation elements (*gharar*). This aligns with the teachings of the Prophet, who forbade any dealings involving goods that could be moved before being received from the seller [33] (Hybrid Contracts). A hybrid contract refers to an arrangement between two or more parties, aiming to execute a transaction that combines two or more distinct contracts (e.g., lease and purchase agreements) with varying features and legal implications, ultimately accomplishing a specific viable outcome. In such agreements, all legal effects and consequences, along with associated rights and obligations, are regarded as a unified whole, akin to the legal effects of a single contract [34].

According to the Council of Islamic Senior Scholars in Saudi Arabia, this approach ensures that the lessee is not burdened with contractual obligations over the lease term that should be the owner's responsibility, which is common in financial leasing contracts. For instance, under the Palestinian Financial Leasing Law No. 6 of 2014, the lessee must cover operational and periodic maintenance expenses and be held accountable for any damage to the leased property, whether caused by negligence or external factors. Additionally, as stated by AAOIFI, the *Ijarah Muntahiyah bi-t-tamlik* is essentially a novel sales contract disguised as a lease, so it does not lead to the consequences of a sale. The seller's ownership persists until the buyer fulfills their obligations to guarantee the financial institution's property rights. As a result, this prevents the simultaneous engagement in two contracts over one property, which goes against Islamic principles [[35], [36], [37], [38]].

However, it is essential to note that scholars have differing opinions about the legality of these contracts. *Abū Ḥanīfah* and *Abū Yūsūf* scholars permit the sale of goods before being received from the initial seller based on *istiḥsān* (juridical preference), supported by several verses related to buying and selling that are applied in a general context. This stance contradicts the opinions of *Muḥammad, Ja'far*, and *al-Shāfi’ī*, who oppose prohibiting selling goods before their acceptance [33]. The concept of hybrid contracts in Islamic finance has sparked controversy due to a particular hadith prohibiting “two contracts in one transaction” (Mālik, Muwatta’, Vol. 2, No. 663, ed. 2005). The misinterpretation of this hadith can undermine any efforts to permit the amalgamation of contracts in Islamic finance, regardless of the specific nature and characteristics of the combined contracts. Consequently, this could impede the development of new products within Islamic banking [34]. Numerous research papers have explored this topic, revealing that many Islamic banking and finance products and services are constructed by combining multiple contracts. Advocates argue that such combinations are permissible in Islamic banking and finance if they adhere to the Shariah guidelines and parameters regarding hybrid contracts. They maintain that it is entirely lawful to structure *Shariah*-compliant products in this manner, provided that the principles of Shariah are rigorously followed throughout the process [34]. Therefore, it came to light that despite encountering some inevitable challenges, the *Ijarah* ending with ownership proves to be a Sharia-compliant agreement, effectively replacing the conventional leasing model in the industry while satisfying customers' needs [39].

In its 50-s session on February 5, 2000, the Council of Islamic Senior Scholars declared the lease-to-own contract invalid if it included the transfer of property ownership through the mere payment of lease installments. This ruling was based on the recognition that combining the sale and lease contracts in a single document is unlawful due to their distinct nature and implications. A sale contract necessitates the complete transfer of ownership and its associated benefits to the buyer. If this transfer occurs before the lease contract is established, it is invalid since the purchaser already owns the property. Conversely, a lease contract entails transferring property benefits to the lessee without them shouldering the responsibilities of its destruction, consumption, or maintenance expenses. The lessor guarantees the leased property, except in cases where the lessee engages in transgression or negligence. This differentiation in nature and obligations between a sale contract and a lease contract was the basis for the Council's decision to invalidate such a combined arrangement [6,19]. Furthermore, the International Islamic Fiqh Academy convened in Riyadh in 2001, declared in its resolution No. 12/4/110 that any agreement automatically transferring ownership to the tenant based solely on the installments paid without needing a new agreement was invalid. The Academy instead endorsed agreements that permit the tenant to utilize the lease by paying a specified fee for an agreed-upon duration, granting the tenant the right to own the property as a gift through the lease if all the agreed installments are duly paid [17].

This approach aligns with the ruling of the “International Islamic Fiqh Council, a subsidiary organ of the Organization of the Islamic Conference,” during its twelfth session in Riyadh, Kingdom of Saudi Arabia, which took place on September 23, 2000. The council's decision validates the existence of two distinct contracts if the sale contract is finalized after the lease term concludes, following the full payment of all rent installments.

As a result, in Arab countries, the legal interpretation categorizes financial lease contracts issued by commercial institutions as contracts for installment sales. The contractual relationship between the parties is governed by the provisions of the sale contract, irrespective of the “named characterization” they may have agreed upon. This legal principle is based on the notion that legal relations should be determined according to the agreed-upon obligations rather than the declared titles [7,40,41]. Once all the responsibilities of the financing institution are transferred to the customer, the contract transforms into a sale, and the ownership transfer must take place as per the agreed terms. This is exemplified in Egyptian law, where if a financial lease transaction effectively transfers ownership, it is deemed a sale, regardless of being labeled as a “lease agreement.” This interpretation is fully in line with the general contract principles in Egyptian law, as specified in Article 430 of the Egyptian Civil Code, which validates the agreement to withhold ownership transfer to the buyer until the full payment is made. Similar provisions can be found in Article 140 of the Kuwaiti Commercial Law No. 68 of 1980.

### 2.2 Requirements for valid *Ijarah Muntahiyah bi-t-tamlik* transactions

In the realm of Islamic banking, Islamic banks often employ *Ijaara muntahia bil tamlik* as a financing product. This involves the Islamic bank leasing an asset to a client, and through this arrangement, the client gains ownership of the asset at the lease period's conclusion and the financing tenor [13]. AAOIFI's standard No: 9, governing *Ijarah* and *Ijarah Muntahia Bittamleek*, is structured into eight distinct sections, each addressing specific aspects of the standard. These sections are as follows [42]: [1] Scope of the standard [2], Obligations regarding the promise to lease an asset [3], Procedures for acquiring the asset or its usufruct by the institution [4], Process of concluding an Ijarah contract and the different forms of Ijarah [5], Matters about the subject of Ijarah [6], Treatment of guarantees and Ijarah receivables [7], Modifications to the Ijarah contract, and [8] Aspects related to the transfer of ownership in the leased property in Ijarah Muntahia Bittamleek.

Certain key elements must be considered in an *Ijarah*. The contracting parties, namely the lessor and lessee, form the foundation. It's possible to involve multiple lessors or lessees in the contract. Additionally, a wakil or agent may be employed to act on behalf of a party. If the asset is co-owned, consent from all co-owners is essential before leasing it. The contracting parties must possess the legal capacity to execute the *Ijarah* arrangement. The second component involves the *Ijab* (offer) and *Qabul* (acceptance) process, establishing the *Ijarah* contract. This can be done orally, in writing, or using any other *Shariah*-approved means. The agreed-upon and Shariah-compliant terms and conditions of the contract bind both parties. The third component pertains to the asset and usufruct [2]. The asset and right to use must comply with *Shariah*, be valuable, existing, identifiable, accessible, deliverable, non-debt-based, and non-perishable. However, these requirements don't apply to assets or usufruct under an *Ijarah* arrangement. Lastly, the rental terms are determined during the establishment of the contract. The parties may agree on cash or in-kind payments, upfront or deferred payments, and fixed rental periods (minimum and maximum). The lessor cannot increase the rental rate unilaterally, but both parties may collectively revise it over time. The lessor can receive and use the rental in advance [15].

Standard No. 9 encompasses numerous legal provisions that regulate the contractual relationship between the parties involved. It covers aspects such as the definition, conditions of validity, composition, consequences, and contract terminations. For instance, Article 4/1/1 within this standard emphasizes maintaining equilibrium in the contractual relationship by prohibiting any clause granting one party the unilateral authority to terminate or modify the contract without the other party's consent. Furthermore, Article 9/7 of the standard disapproves of a sale contract that involves transferring ownership at a future date concurrently with concluding a lease contract in a single transaction. Additionally, Article 9/1 mandates the parties to agree upon the tenant's ownership of the property through a separate document from the lease agreement. This ensures that the lease provisions appropriately apply to the contractual relationship during the installment payment phase (as per International Islamic Fiqh Academy Resolution No. 13 (1/3)).

From an Islamic perspective, leasing can be classified into two main categories based on its nature. The first category involves leasing materials, where individuals pay for using certain materials others own for a known value. This can pertain to immovable objects such as transportation and clothing or fixed objects like real estate, premises, and lands. The second category is known as “leasing of work,” which entails hiring someone to perform a specific task in exchange for a known wage (Al-Momani, 2015, p. 194). Following AAOIFI's standard No. 9, Article 9/7 presents various approaches for transferring ownership, aligning with the evolving trends of Islamic transactions jurisprudence. These methods are devised to facilitate the transfer of property in a manner that complies with Islamic principles and practices (Abu Hamdiya 2019; Alkhan 2020; Ibrahim 2005; Ismail 2001; Sifat and Mohamad 2016):

1 A promise to sell at a nominal price, a real price, or a price equivalent to the rent for the remaining period.
2 A donation promise [2].

This promise must be documented separately and contemporaneously with the lease agreement [16]. Also, the transfer of ownership should be organized in an independent document from the lease contract. For this reason, Article 8/3 stipulates that: “in cases of ownership by way of a promise, a new contract must be concluded to transfer the ownership.”

3 Ownership transfer under a donation contract is contingent upon the payment of installments. Once all installments have been fulfilled, ownership shall be transferred without needing a new contract (as per Article 8/4).

In contrast to prevailing trends in Islamic jurisprudence, the AAOIFI standards acknowledge the validity of a unilateral promise based on the opinions of certain imams (jurists), including *Imam Malik, Ibn Taymiyyah*, and *Ibn al-Qayyim al-Jawziyyah*. These imams concur that suspending the sale contract by fulfilling a future condition is acceptable as long as it serves the legitimate interests of the contracting parties and is in line with the teachings of the Qur'an and the Sunnah of the Prophet [35,43]. According to this perspective, the starting point for contractual conditions is permissibility unless a specific legal prohibition exists. In other words, any contractual condition or term that God and His Messenger do not explicitly forbid in the context of contracts is considered permissible and valid [44]. Also, this standard explicitly states that the lease contract provisions must govern the relationship throughout the lease term (as per Islamic Fiqh Council Decision No. 13 (1/3)). It is not permissible to introduce any conditions that contradict the requirements of the lease contract, as the essence of the contractual relationship is akin to an installment sale or a sale contingent on the full payment of rental dues [12,29,45]. The purpose of this provision is to safeguard the tenant's interests and prevent them from being burdened with contractual obligations that are typically the responsibility of the seller, who is the original owner of the leased property, throughout the duration of the lease agreement [24,46].

## 3 Research methodology

This research is the first study to examine the legitimacy of the *Ijarah Muntahiyah bi-t-tamlīk* contract terms issued by Islamic banks in Palestine. It introduces practical recommendations for the best practice of these transactions to be consistent with mandatory rules. Until now, no specialized legal studies have examined the extent of these contracts' compatibility with the relevant legal standards. Previous research results were limited to some accounting studies that examined the contracts' compatibility with accounting standards from an accounting point of view. Therefore, this research followed a comparative analytical descriptive approach to examine the legitimacy and compatibility of *Ijarah Muntahiyah bi-t-tamlik* contracts issued by Palestinian Islamic banks with the applicable legal standards.

The research utilized some *Ijarah Muntahiyah bi-t-tamlik* contracts conducted by Islamic banks operating in Arab countries (Jordanian Islamic Bank and Kuwait Finance Bank) to benefit from their experiences. Islamic Banks received AAOIFI appreciation in many periodic reports issued by the organization in the last ten years are used in this research.

The research methodology involved the following steps:

• Literature review: An extensive literature review explored existing studies on *Ijarah Muntahiyah bi-t-tamlik* contracts and their compliance with Islamic standards and legal regulations. Any accounting studies that had examined the contracts from an accounting perspective were identified.
• Data collection was gathered from Palestinian Islamic banks that offered *Ijarah Muntahiyah bi-t-tamlik* contracts. Relevant documents, agreements, and contract terms were obtained to analyze their structure and provisions.
• Comparative analysis: The collected data from Palestinian Islamic banks was compared with the *Ijarah Muntahiyah bi-t-tamlik* contracts of Jordanian Islamic Bank and Kuwait Finance Bank to draw insights from their experiences and best practices.
• Shariah compliance assessment: Data collected were utilized to assess the contracts' conformity with AAOIFI standards and Islamic principles.
• Legal framework analysis: The relevant legal framework in Palestine, including *Al-Majjahllah* Code, was analyzed to identify any discrepancies and challenges in implementing these contracts.
• Practical recommendations: Based on the research findings, practical recommendations were formulated to ensure the legitimacy and compliance of *Ijarah Muntahiyah bi-t-tamlik* contracts with Islamic standards and mandatory rules in Palestine.
• Conclusion: The research findings were summarized, emphasizing the significance of this study as the first specialized legal examination of *Ijarah Muntahiyah bi-t-tamlik* contracts in Palestine. The practical recommendations were reiterated, and potential avenues for future research in this area were proposed.

## 4 Results and discussion

The results of the field research pinpointed two distinct challenges that impede the effective implementation of the PMA decision. The first challenge scrutinizes the congruence between Islamic banking principles and the foundational tenets of contract law enshrined in national regulations. Specifically, the legal recognition of unilateral promises as a viable source of binding obligations presents a critical concern. As these promises are central to lease-to-own contracts, their lack of legal value under conventional contract law can raise complications for Islamic banks. The paper thoroughly examines the implications of this disparity and explores potential solutions to align these contracts with Islamic banking principles and legal requirements. The second challenge, also unveiled through field research, revolves around Islamic banks' prevalent adoption of pre-drafted contracts. As brought to light by the field research, this practice has the potential to disproportionately allocate responsibilities to customers, thereby potentially conflicting with the fundamental ideals of Islamic finance that underscore the equitable and balanced distribution of obligations among the various parties involved in a financial transaction.

### 4.1 Challenges in regulating the Ijārah Muntahiyah bi-*t*-tamlīk in Palestine

**Fig. 1:**
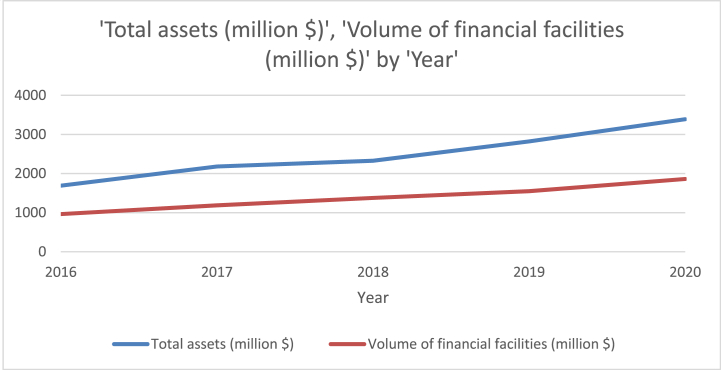
Total assets of Islamic banks in million dollars. Source: Palestinian Monetary Authority, Annual Report 2020.

In Palestine, Islamic banking is currently practiced by three banks: The Arab Islamic Bank, established in 1995; the Palestinian Islamic Bank, established in 2013; and Al-Safa Bank, established in 2016. Despite their limited number among the thirteen banks in the region, these Islamic banks have experienced notable growth in their net assets. Specifically, in 2020, they achieved a remarkable growth rate of 20%, reaching a total of $3.4 billion in net assets, compared to $2.8 billion in 2019. Furthermore, the total assets held by Islamic banks accounted for 18% of the entire Palestinian banking sector. Over the period from 2016 to 2020, these Islamic banks experienced a steady growth rate of 5% in total assets. Another indicator of their expanding influence is the substantial increase in direct financial facilities offered by Islamic banks. From 2016 to 2020, the value of these facilities surged by an impressive 93%, rising from $965.445 million to $1860.831 million (Fig. 1)

A noteworthy point is that Palestinian Islamic banks offer *Ijārah Muntahiyah bi-t-tamlīk* transactions exclusively for real estate properties registered in the Land Registry Departments. This preference is due to the stable presence and consistent values of such properties in the Palestinian market. These transactions account for approximately 10% of the total financing provided by the Palestine Islamic Bank and 6% of the total funding offered by the Arab Islamic Bank [16,47]. Despite engaging in numerous *Ijārah Muntahiyah bi-t-tamlīk* transactions, these banks employ standard contracts, which outline the operational procedures of the agreements as follows:

• The client initiates the contracting process with the bank, expressing their intention to purchase a specific property for their personal use.
• Both parties enter into a lease-promise agreement, wherein the client commits to renting the designated property according to the principles of *Ijārah Muntahiyah bi-t-tamlīk* for a predetermined period, with a known total amount representing the installments to be paid to the bank. Additionally, this agreement includes a condition stipulating that the bank will transfer ownership of the leased property to the lessee at no cost within thirty days from the final installment payment date.
• Subsequently, using a standard form, the two parties finalize an *Ijārah Muntahiyah bi-t-tamlīk* agreement.
  • Upon the tenant fulfilling all agreed-upon installments, the bank transfers ownership of the property to the client, free of charge, per the donation promise, before the competent authorities.

This agreement clearly demonstrates that the *Ijārah Muntahiyah bi-t-tamlīk* transaction operates on a unilateral promise basis, wherein the agreement utilizes the phrase “the first party promised the second party.” The lease agreement is also titled “a promise of rent.” Furthermore, Article 5/19 of the Tenancy Promise Agreement specifies that the “first party” (referring to the owner) pledges to the “second party” (the lessee) the transfer of property ownership within a maximum of thirty days, provided the tenant fulfills all the agreed installments throughout the lease term.

The primary issue faced by this organization is the ongoing disagreement among Muslim Scholars regarding the concept of the unilateral promise [44]. While there is a consensus among most that fulfilling promises is praiseworthy, many contend that it is not legally obligatory from a judicial standpoint, even though it is considered a religious duty rooted in kindness and charitable conduct [48]. Following the Almighty's words, “No ground of complaint against the doers of good,” *El-Maliki* jurisprudence dictates that promises must be honored if they are contingent upon a condition, and AAOIFI standards also adopt this principle. As financial transactions evolve and society increasingly relies on banking activities, considering promises as legally binding obligations falls under the purview of financial rights derived from Islamic Sharia and the law, as emphasized by certain imams in Fiqh [6]. Therefore, the leasing promise is deemed permissible when it involves the financial institution undertaking the purchase and delivering the specified item to the agreed-upon location for the lease contract [46]. According to the perspective presented, the International Islamic Fiqh Academy, in its No. 40–41 (2/5 and 3/5) resolution, determined that this type of promise elevates to the level of a formal contract even before ownership is transferred, thereby attributing both legality and obligation to it simultaneously.

According to clause 9/5 of the AAOIFI Shariah standard on Ijarah financing, a significant interval of time must pass between the two transactions of sale and leaseback. In other words, when one party purchases an asset and subsequently leases it back to the same party based on *Ijarah Muntahia Bittamleek*, a reasonable period must be elapsed between the sale and leaseback operations. This time-lapse allows for potential changes in either the asset itself or its value, as specified in clause 9/5 of the AAOIFI Shariah standard: 9 on *Ijarah* financing [42]. The inclination of modern Islamic jurisprudence towards considering the unilateral promise as legally binding represents a prevailing scholarly trend. However, the texts of Al-Majalah do not concur with this perspective and instead align with the traditional approach of jurisprudence. As a result, this agreement process gives rise to certain legal issues, which will be elaborated upon in this study section.

#### 4.1.1 Lack of legal effect for the unilateral promise

*Al-Majjahllah* Code, which adheres to al-Hanafi jurisprudence principles, does not recognize the validity of suspending a sale based on a condition or future event, nor does it consider the unilateral promise as legally binding [28,44,48,49]. Article 171 of *Al-Majjahllah* states: “In cases of a reception formula, which involves promises like ‘I will sell’ or ‘I will buy,’ the sale does not become valid.” Most Jurists do not consider the unilateral promise valid, as it is not allowed to make contracts of financial exchanges conditional on specific terms. The reason behind this is that the legal effect of a sale is the transfer of ownership, which should occur by the operation of law and not be subject to the parties' will within the contract [50]. While Islamic law emphasizes the importance of fulfilling promises, jurisprudence regarding financial transactions views it as a moral obligation rather than a legal requirement. In other words, although it is considered morally binding to honor promises in financial matters, neither the promiser nor the judge is legally obligated to enforce its fulfillment [51,52].

Islamic jurisprudence agrees that, as a general principle, suspending obligations based on future conditions is not permissible. However, there are certain contracts where this practice is considered lawful, creating exceptions to the general rule [28,48,49,53]. The Explanatory Memorandum of *Al-Majjahllah*, as presented by the scholar *Ali Haydar*, clarifies that suspending obligations is permissible only in specific situations in accordance with Islamic rules. For instance, it is valid in contracts such as attorneyship, agency agreements, and wills. However, certain contracts, like sales, rent, and gifts, cannot be subject to suspension. An example given in *Al-Majjahllah* is that if someone says, “If so-and-so returns from his travels, I will sell you my house for this price,” this agreement is not considered valid. The reasoning behind this is that the condition may or may not be fulfilled, and the sale is linked to uncertain factors like existence and non-existence. Islamic scholar *Al-Nawawi* also emphasizes that suspending a sale on a future condition, like saying, “I will sell you this house if it rains,” is not permissible and is considered void. This prohibition is based on the principle of avoiding uncertainty or risk (*al-gharar* sales), which the Prophet Muhammad, peace be upon him, forbade. Furthermore, this type of transaction goes against the pillar of consent, which is a fundamental aspect of any contract. According to Muslim jurist *Al-Qarafi*, the transfer of ownership in contracts depends on mutual consent, which requires certainty and avoids uncertainty, as it cannot be guaranteed to occur. Such suspensions contradict the very purpose of a contract, which is to transfer ownership once an agreement is made.

Conversely, certain modern interpretations of the Al-Majjahllah Code contend that Article 84 validates unilateral promises under specific conditions, binding them once the stated condition is fulfilled. This article explicitly states that any promise hinging on a condition becomes irrevocable when that condition is met. However, despite this provision, the Palestinian judiciary has consistently favored the initial approach, which does not compel the enforcement of promises in contracts. As a result, if an offer and acceptance meet the requirements of a unilateral promise according to Article 84, the promisor's failure to fulfill the promise cannot be legally enforced by the promisee.^1^ Hence, if the offer and acceptance fulfill the criteria of a unilateral promise, and the promisor fails to uphold their commitment, the promisee lacks the legal authority to compel the promisor to carry out the promised action.^2^ Even though the promisee cannot force the promisor to fulfill the promise in the context of a unilateral promise, if the promisee suffers harm due to the promisor renouncing the promise, they may have the right to seek compensation for the damages incurred. This compensation can be pursued under tort liability rules, which deal with civil wrongs and the resulting liability for the harm caused to another party. In such cases, the promisee may be entitled to seek redress for the losses or damages they have suffered due to the promisor's failure to fulfill their promise [54].

In the context described, it is important to note that the legality of unilateral promises cannot be invoked in most Arab countries, including those influenced by Islamic law. These countries have taken steps to codify their civil codes in recent decades and have explicitly recognized the validity of unilateral promises as binding sources in financial transactions. For instance, Article 133 of the UAE Civil Transactions Law states that a future tense expression denoting an absolute promise may constitute a binding contract if the parties involved intend it to be so. Similar provisions are also present in the civil codes of other Arab countries, such as Iraq, Egypt, Syria, Kuwait, Bahrain, and Qatar, which validate the enforceability of unilateral promises when the contracting parties manifest their intention to be legally binding. Moreover, the Explanatory Memorandum of the UAE Civil Transactions Law underscores the validation of situations necessitated by the requirements of commercial dealings, even though such situations are not permitted under *Al-Majjahllah*. According to this modern law, when a promisor expresses a unilateral promise, they become legally obligated to fulfill it if the promisee intends to accept the offer. Furthermore, the Palestinian Civil Code's Draft follows a similar approach, aligning with contemporary legislative trends and including a provision (Art. 90) that recognizes the validity of unilateral promises in financial transactions.

#### 4.1.2 Lack of legal effect for suspending donation on a future condition

The legality of a donation suspension agreement, which depends on fulfilling a future condition, is a matter of ongoing legal debate. Most jurists, including those from the *Al-Hanafi, Al-Shafi'i*, and *Al-Hanbali* schools of jurisprudence, do not consider such agreements valid. Their reasoning is grounded in the general principle that agreements should be enforceable without ambiguity or uncertainty [46,48,53]. In his book *Badaa’ al-Sana'i* (6/184), the Muslim jurist *al-Kasani* further emphasizes that the donation contract involves the transfer of ownership, making it impermissible to suspend such a contract on a future condition that may or may not be realized. This underlines the principle that a donation, being a transfer of ownership, should not be subject to uncertain or contingent conditions [19]. Indeed, some scholars validate the suspension of donation contracts based on their opinion that unilateral contracts have a wider scope than bilateral contracts. They argue that the Prophet Muhammad (peace and blessings of Allah be upon him) set an example of this by donating a gift to the king of Abyssinia, with the condition that the gift would be effective only if the king were alive when the messenger delivered it to him. In this context, these scholars believe that unilateral contracts can accommodate certain conditional arrangements, like the one observed in the Prophet's donation, where the fulfillment of the condition does not rely on the actions of both parties involved. However, it is essential to note that this perspective is a matter of scholarly interpretation and not unanimously agreed upon by all Islamic jurists [43,46,51,53,54].

The texts of *Al-Majjahllah* support the first approach, which does not uphold the validity of such agreements. According to *Al-Majjahllah*, the donation agreement's validation relies on the transfer of ownership and receipt of the contract's subject matter, as stated in Article 57. This article states, "*A gift becomes absolute only when delivery thereof is taken*. *A person bestows a thing upon another person by way of gift. Such gift is not binding until delivery thereof has been taken*”. The Explanatory Memorandum adds that the donation does not take place by mere promise, as if someone says to another: “*I will give you this money, and he did not give it, so the person who made the promise, in this case, cannot be forced to fulfill what he promised*” [55]. Also, Article 854 of *Al-Majjahllah* invalidates any donation added to the future. It states, "*A donation which is to take effect in the future is invalid*. For example, suppose a donor states that he has made a gift of a certain thing with effect from the first of next month. In that case*, the gift is invalid*.” Also, Article 862 of *Al-Majjahllah* expressly grants the donor the unconditional right to “revoke the gift of his own accord before delivery thereof is taken” [52,56].

Finally, it is important to highlight that the Draft of the Palestinian Civil Code takes a distinct approach compared to *Al-Majjahllah*. Article 525 enforces the donation agreement once there is a mutual agreement between the parties, without necessitating the transfer of ownership. Additionally, Article 522 validates the conditional donation of a specific action or work to be performed by the recipient. Article 526 further states that the donor is obliged to deliver the gifted item to the donee, with the provisions concerning the delivery of a sold item being applicable in this regard.

#### 4.1.3 Invalidity of unregistered real estate transactions

For any real estate transaction to be considered valid, it must be registered with the Land Registry Department, as stated in Article 16/3 of the Land Settlement Law and Article 2 of the Law on Disposal of Immovable Property No. 49 of1953.^3^ This requirement applies to both sale contracts and unilateral promises. Consequently, the Court of Cassation ruled against the validity of a unilateral promise made by the landlord to transfer real estate ownership to the purchaser since it had not been registered with the Land Registry Department [44,54].^4^

The Draft of the Palestinian Civil Code acknowledges the significance of registering real estate transactions. Its explanatory memorandum clarifies that although a unilateral promise is a consensual agreement, if the contract's conclusion necessitates a specific formality, such as a mortgage contract or a company's articles of incorporation, then the promise must adhere to the same formality. Failure to comply with the required formality renders the unilateral promise null and void. However, despite this invalidity, certain legal consequences can still be arranged, and compensation may be claimed if all liability conditions are met.

### 4.2 Consistency of *Ijārah Muntahiyah bi-t-tamlīk* contracts terms with the AAOIFI standards

The first part of this research demonstrated that the AAOIFI standards strive to enforce the lease contract terms concerning the interaction between the financing institution and the client. This ensures that the landlord's legal responsibilities are not passed on to the tenant during the lease's validity period. As the standard contracts issued by Islamic banks in Palestine explicitly state that their terms must comply with the Islamic *Shari'a* standards and the general regulations in Palestine, it becomes essential to assess whether such compatibility exists.

It is essential to highlight those numerous standards released by the AAOIFI and decisions made by the International Islamic Fiqh Academy stress the importance of adhering to the lease provisions throughout the entire duration of the contractual relationship. This requirement must not be disregarded or violated because the property was acquired based on a promise from the landlord that the tenant would eventually become the owner, and the ownership would transfer to the tenant upon the agreed lease term's completion [56,57]. One of these texts is the decision emphasizing the importance of applying leasing provisions, specifically regarding the owner's responsibility for basic maintenance and insurance premiums. This decision was made during the first jurisprudence symposium of the Kuwait Finance House held in Kuwait in May 1987. Additionally, the decision issued by the International Islamic Fiqh Academy, which resulted from the twelfth session of the organization of the Islamic Conference held in Riyadh, Kingdom of Saudi Arabia, on September 23, 2000, with reference number 110 (12/4), declares any agreement that violates this ruling as invalid [57].

Also, the standard contracts explicitly state the obligatory nature of adhering to these general provisions (Islamic standards and national law) in cases where the Agreement does not make any specific provisions. This implies that these contracts acknowledge the authority of the contracting parties to agree on terms that may contradict Islamic standards and general regulations. Islamic banks rely on such contractual terms when dealing with customers, disregarding their invalidity. Consequently, numerous violations become evident, and these violations are thoroughly discussed in this section of the study.

#### 4.2.1 Tenant's obligation to make the property fit for the purpose intended for

Article 5/4 of the standard contracts impose the tenant's responsibility to fulfill all requirements for obtaining permits and licenses necessary for using the leased property and ensure access to essential services like water, electricity, telephone lines, sewage, etc. Additionally, Article 5/7 requires the lessee to bear all expenses, costs, fees, taxes, fines, and other obligations related to the leased property, whether legally owed by the owner or the lessee, without the right to seek reimbursement from the owner. If the lessee fails to fulfill these obligations, the owner can perform them at the lessee's expense. Moreover, Article 5/11 denies the tenant the right to claim reimbursement from the landlord for any amounts spent by the tenant to complete or improve the leased property or its common areas. It is evident that these conditions directly contradict many of the Islamic standards issued by the AAOIFI and the provisions of the lease contract provided in *Al-Majjahllah.* For instance, Article 5/2/2 of the AAOIFI standards considers the rent due only for the benefit received, not simply by entering the contract. Similarly, Article 4/1/3 of the AAOIFI standards does not validate any contractual condition obliging the tenant to pay rent for periods during which the landlord delayed preparing the property for use according to the intended purpose. In such a case, the tenant's rent should either be reduced by the property's value during the non-utilization period, or the tenancy period should be extended by a period equivalent to the non-utilization period.

The texts of *Al-Majjahllah* and the AAOIFI standards are in complete agreement. For instance, Article 529 of *Al-Majjahllah* explicitly stipulates that the landlord is responsible for covering all expenses required to ensure that the leased property is suitable for its intended purpose. If the landlord unreasonably declines to fulfill this duty, the tenant can undertake the necessary improvements at the owner's expense. Furthermore, Article 478 of *Al-Majjahllah* relieves the tenant from paying rent if they cannot derive any benefit from the property [58]. In the case of a partial loss of that benefit, the lessee is entitled to deduct a portion of the rent equal to the value of the lost benefit. The rationale behind these provisions is that the rent payment in the lease contract is a consideration for the benefit received by the tenant. As a result, the tenant cannot rightfully claim this rent unless the owner fulfills their obligation to provide the tenant with this benefit [59]. Furthermore, the lessee's ability to utilize the leased property is inherently tied to receiving the property in a condition suitable for the intended purpose for which it was prepared. Therefore, this responsibility should fall upon the property owner [41,50].

Moreover, the notion of delivery is not confined solely to the main lease but also encompasses any additional structures or annexes intended to serve the leased property. These annexes are determined based on the agreement between the parties or the nature of the leased property (such as the garage, water, electricity, gas, heating, air-conditioning equipment, etc.). The essential condition is that both the leased premises and their corresponding accessories are useable and capable of fulfilling the intended benefit they were prepared for [54]. This obligation further encompasses any contractual expenses, if applicable, such as registration fees, before the competent authorities. According to AAOIFI, these expenses will be shared equally between the contracting parties, as both parties derive financial benefits from the contract [47]. These works represent essential and non-operational expenses crucial for the lease's validity and usability, as the lessee cannot benefit from the lease without them. Consequently, the agreement compels the tenant to bear these expenses, potentially violating the AAOIFI standards, except when the tenant performs the works at the owner's expense. The tenant's obligation to cover these expenses may lead to a convergence between characterizing the relationship as a lease or an installment sale [60].

In addition, the standard contracts categorize property insurance fees as operating expenses and require the lessee to pay them on behalf of the lessor [61]. This provision contradicts the ruling of the Council of the International Islamic Fiqh Academy, originating from the twelfth session of the organization of the Islamic Conference held in Riyadh, Kingdom of Saudi Arabia, on September 23, 2000. The ruling explicitly states that if the lease contract includes property insurance, the insurance must be of the Islamic cooperative type, not commercial, and the responsibility for bearing the insurance expense lies with the landlord, not the lessee [16]. Consistent with AAOIFI standards, the ninth clause of the *Ijārah Muntahiyah bi-t-tamlīk* contract, issued by the Jordan Islamic Bank, affirms the owner's prerogative to secure the lease according to their discretion. However, the expenses of this insurance are borne by the owner as the rightful owner of the property. Similarly, Article 17 of the contract issued by the Kuwait Finance Bank also includes the same provision.

#### 4.2.2 Tenant's obligation to pay all maintenance expenses

*Ijarah* entails a contractual arrangement wherein one party grants a specific benefit to another party (the beneficiary) for a predetermined duration. In return for accessing this benefit, the beneficiary is obligated to provide compensation to the granting party. Since *Ijarah* is centered around a lease-based transaction, it typically establishes a lessor and lessee relationship [13]. There is an agreement between scholars that the lessor assumes full responsibility for any damage, repairs, insurance, and depreciation of the leased asset [62]. In contrast, Article 5/5 of the standard contracts imposes on the tenant the duty to perform periodic maintenance as requested by the bank throughout the contract term. However, this condition conflicts with the landlord's responsibility to ensure the tenant's ability to benefit from the leased property, necessitating the landlord to undertake all necessary repairs to maintain the property's validity for the intended benefit throughout the lease period [24,63]. Hence, this contractual provision contradicts AAOIFI standards, which prohibit the landlord from requiring the tenant to ensure essential property maintenance, as the benefit continuity depends on it. Nevertheless, these standards require the tenant to cover the operating maintenance expenses (Article 5/1/7). If the benefit is partially affected due to the tenant's actions, the tenant is responsible for its restoration, and the rent will not be reduced for the missed benefit during that period (Article 5.1.6). These principles align with the decision of the Council of the International Islamic Fiqh Academy, derived from the Organization of the Islamic Conference in its twelfth session held in Riyadh, Kingdom of Saudi Arabia, on September 23, 2000. The decision places the guarantee of the leased property on the owner's shoulders, not the lessee's. Therefore, the lessor bears the responsibility for any harm that befalls the property not caused by the tenant's negligence or default, and the lessee is not obligated to take any action if the benefit is not realized [38].

In this regard, the AAOIFI standards distinguish between the basic maintenance expenses and the operational maintenance expenses as follows [6,17,19]:

A- Basic maintenance expenses protect the leased property from deterioration and ensure its continued existence and desired functionalities. These expenses include maintaining air conditioning, central heating, addressing water leaks, and caring for shared amenities such as swimming pools and elevators. The responsibility for covering these maintenance costs lies with the owner of the property [54].

B - Operational maintenance expenses cover the upkeep and replacement of components that may experience wear and tear from everyday use, such as repairing faulty power lights and water taps. The tenant is responsible for covering these maintenance costs [46]. It is noteworthy to mention that both the provisions of Islamic jurisprudence and the texts of *Al-Majjahllah* (Articles 481, 530, and 532) offer identical provisions [7].

As stipulated in clause 5/1/8 of the AAOIFI Shariah standard governing Ijarah financing, the lessor can obtain permissible insurance on the asset whenever feasible. Alternatively, they may assign the responsibility of securing insurance to the lessee, with the expenses borne by the lessor. Therefore, for conducting Ijarah transactions, Islamic banks are generally not obligated to acquire insurance coverage unless regulatory authorities require it. However, if they do opt for insurance, it must be sourced from permissible avenues. Specifically, Islamic banks must obtain insurance from takaful operators [42]. In alignment with AAOIFI standards, the eighth clause of the Ijārah Muntahiyah bi-*t*-tamlīk contract issued by the Jordan Islamic Bank restricts the tenant's responsibility to perform operational maintenance exclusively. Basic maintenance tasks are the obligation of the owner. A similar provision is outlined in the tenth clause of the contract issued by the Kuwait Finance Bank. 10.13039/100014337Furthermore, these clauses grant the owner the authority to inspect the leased property to ensure its appropriate utilization and confirm that the tenant has not violated or neglected its proper use.

#### 4.2.3 Lessee's obligation to bear the loss of the leased property

Article 5/5 of the standard contracts mandates the tenant to be responsible for the security and safety of the leased property, its accessories, and utilities. Additionally, the tenant bears criminal and civil liability arising from any damages that may occur to the property or third parties. This provision directly contradicts the essence of a lease contract, which typically requires the lessee not to assume such guarantees except for those resulting from their actions, negligence, or the actions of their subordinates, specifically affecting the leased premises [7]. Hence, if the leased property is damaged due to a cause beyond the lessee's control, and the lessee can demonstrate that they have taken adequate measures to safeguard it, the lessor will be responsible for covering the incurred expenses [43]. Contracts issued by other Islamic banks in Jordan and Kuwait adopt a different approach, incorporating a similar provision. For instance, the seventh clause of the contract issued by the Jordan Islamic Bank states that the lessor assumes liability for any loss from the moment they receive the leased property, except in cases of force majeure or circumstances beyond their control. On the other hand, the lessee will be held liable for any total or partial loss affecting the property only if it results from infringement, failure to use it, or breach of the duty to maintain it. The contract issued by the Kuwait Finance Bank contains the same provision.

Requiring the tenant to assume responsibility for the damages resulting from the destruction of the leased property directly contradicts the AAOIFI standards and the general provisions outlined in Al-Majjahllah. There is an agreement between scholars that the lessor bears the risk associated with the uncertainty surrounding the useful life of the leased asset [62]. They also concur that the leasing object should not be guaranteed against destruction, as this responsibility falls on the tenant. However, the tenant can be held accountable in cases of misconduct or negligence [2].

For instance, Article 7/1/4 of the AAOIFI standards stipulates that the leased property is entrusted to the lessee, and the lessee is not held liable for it, except in cases of transgression or negligence on their part. Article 5/1/8 obliges the lessor to ensure the leased property throughout the lease period, unless there is a breach or negligence on the part of the lessee. To maintain the equilibrium in the contractual relationship, Article 5/1/4 requires the tenant to use the leased property for its intended purpose, refraining from causing intentional damage or negligence in its usage. Furthermore, Article 8/8 states that in the event of a total loss of the leased property, the financial institution bears the difference between the rental amounts paid by the lessee and the market value of the rent on the due date, subsequently refunding this difference to the lessee. In case of destruction of the leased property, the contract is terminated, and no further installments are required (Article 7/1/3). In the case of partial loss, the lessee has the option to terminate the contract or agree with the lessor on a reduced rent, and no rent is charged for the period of lost benefit (Article 7/1/5). Under no circumstances can the lessor exempt themselves from any defects that hinder the tenant from benefiting from the leased property. Additionally, they cannot stipulate that they are not responsible for any defect affecting the property's benefit, whether due to their actions or due to circumstances beyond their control (Article 5/1/5). Moreover, Decision No. 13 (1/13) issued by the Islamic Fiqh Council also ruled on the owner's responsibility (the bank) for losing the leased property unless it resulted from the tenant's negligence. Similarly, *Al-Majjahllah* includes similar provisions (Articles 514–519).

#### 4.2.4 Imposing financial sanctions on the promisor if he breaks his promise

The fourth provision in the standard contracts imposes an obligation on the tenant to pay an upfront amount known as the “earnest money” to demonstrate their commitment to renting the specified property. Should the tenant abandon the lease agreement, they forfeit the right to have this amount refunded. The earnest money compensates the bank for the damages incurred due to the tenant's failure to uphold their promise to rent the property. Moreover, the bank is entitled to seek additional compensation equal to the proven damages if they surpass the amount of the paid earnest money. However, this penalty is not present in the contracts issued by the Jordan Islamic Bank and the Kuwait Finance Bank. Instead, these banks reserve the right to file a lawsuit against the promisor and demand compensation equal to all the damages they incurred as a result of the failure to fulfill the promise in accordance with the general rules (Article 27 of the contract issued by the Jordan Islamic Bank and Article 24 of the contract issued by the Kuwait Finance Bank).

The penalty clause represents a contractual agreement to determine the compensation the creditor will receive if the debtor fails to fulfill their obligation [64]. It should be agreed upon before the breach. If it is agreed upon after the breach, it shall be a reconciliation between the parties on the amount of compensation due [65]. It is usually monetary compensation. It can also be by deducting a sum of money from the value of the contract or paying financial dues that are not yet due [66]. While all Civil Codes in Arab Countries acknowledge and validate the inclusion of penalty clauses in contracts, there are variations among them in terms of how they calculate and determine the compensation in such situations [7,52]. For instance, in the Egyptian Civil Code, Article 224 establishes the penalty clause as conclusive evidence of the compensation owed to the aggrieved party, relieving the creditor of the need to prove the actual damages incurred. However, if the debtor seeks to challenge the agreed amount, the burden of proof shifts to them. Additionally, Egyptian law grants the judge the authority to reduce the value of the penalty clause upon the debtor's request but does not allow an increase at the creditor's request. Syrian law follows a similar approach to that of Egyptian law. On the other hand, Jordanian law grants the judge the discretion to either increase or decrease the amount of the agreed penalty clause upon the request of either party to the contract to align it with the actual damages suffered by the creditor (Article 346). Islamic Fiqh approaches distinguishes between two types of conditions as follows [16,19,67,53]:

1 Penalty clauses that increase the debt owed because of the debtor's delay in implementing his obligation: Islamic law does not permit these conditions because they are a form of usury (*Riba*) [54]. Therefore, the decision of the Islamic Fiqh Academy emanating from the organization of the Islamic Conference in its twelfth session in Riyadh No. 85 of 2000 prohibited this form of a penalty clause. Likewise, the Council's decision regarding installment sales No. 51 (6/2) invalidated it either. It ruled that: “If the purchaser delays in paying the installments after the specified date, it is not permissible to obligate him to any increase in the debt because that is forbidden usury” [16].

2- Penalty clauses that do not increase the debt due but rather compensate the creditor for his damages aim to compensate the creditor for damages caused by the failure of the debtor to perform its contractual obligation. A clear example is the contractor's delay in completing the construction on the agreed date and the supplier's delay in supplying the goods on the agreed date. In these cases, the creditor can deduct part of the financial receivables imposed on him equal to the damage suffered. These conditions are legalized according to the trends of Islamic contemporary jurisprudence but considering two restrictions: the first is that they are commensurate with the value of the damage suffered by the creditor, and the second is the absence of an excuse for the debtor to prevent him from carrying out the obligation. The Islamic Fiqh Academy implemented these restrictions in its resolution No. 65 (7/3) related to the manufacturing (*Istisna’*) contracts, which stated: “It is permissible for the *Istisna’* contract to include a penal condition unless there are force majeure circumstances” [46].

Modern Muslim jurists unanimously agree on the prohibition of tenants demanding an advance payment to be paid to the lessor during the contracting process. This payment is not an advance fee or a guarantee of payment but rather a method of coercing the tenant into fulfilling their promise to rent. As a result, it is deemed an unlawful and coercive penalty [43,51,53,68]. As per clauses 6/3 and 6/4 of the AAOIFI Shariah standard governing Ijarah financing, Islamic banks are not permitted to levy any additional charges in the event of delayed payment of rentals by the customer. However, suppose the delay is not justified by a valid reason acceptable under Shariah principles, and the customer is capable but unwilling to make the rental payments. In that case, as outlined in the contract, the customer must make a charitable contribution as specified when signing the Ijarah contract. A thorough examination of clauses 6/3 and 6/4 reveals that in the scenario where the customer fails to make the rental payments without a valid Shariah-compliant reason, they are obligated to make a charitable donation to designated organizations. Consequently, the amount received by the bank in such cases cannot be recognized as part of the bank's income [42].

Under Palestinian law, *Al-Majjahllah* encompasses several provisions concerning civil liability resulting from contract breaches. For instance, Article 19 states that “injury may not be met by injury, no harm.” Article 20 emphasizes the need to remove any harm caused, while Article 92 establishes that a person who unintentionally performs an act is liable for any resulting loss. Consequently, contractual liability arises when a party fails to fulfill their contractual obligations, and the creditor must prove both the breach and the extent of the resulting damage. The injured party must demonstrate the debtor's breach of the contractual obligations and then establish the amount of damages suffered, regardless of any prior understanding about compensation amounts. Notably, Palestinian law does not address penalty clauses, rendering such agreements legally ineffective since they contradict general legal principles.

As evident from the wording in the standard contracts, the penalty clause appears to serve as a threatening fine rather than a compensation clause. This is evident from the clear indication that the bank reserves the right to demand an increase in the compensation amount from the customer, while the text does not grant the customer the right to seek a reduction equivalent to the actual damage suffered by the bank due to the customer's failure to fulfill their commitment. Such a threatening fine contradicts the principles of Islamic jurisprudence, which prohibit requiring compensation from a debtor for delays in fulfilling their obligations. This prohibition is explicitly addressed in the provisions of the second standard issued by AAOIFI under the title “Debtor in Delay.” This prohibition is because Islamic jurists consider such conditions akin to apparent usury [3,69]. Hence, these contractual terms stand in opposition to the principles of Islamic law. The ruling of the International Islamic Fiqh Academy is clear on this matter: “It is not permissible in Islam to include clauses that impose compensation for delayed payments.” Such penalty clauses are not applicable in cases of delayed debts and enforcing them directly or seeking judicial intervention to implement them would violate the standards of Islamic Sharia.

## 5 Conclusion

The research primarily focused on analyzing the *Ijārah Muntahiyah bi-t-tamlīk* contracts issued by Islamic banks in Palestine. It uncovered several challenges faced by these banks in adhering to the PMA's decision No. 15/2019, which aimed to implement Islamic standards in their transactions. The study highlighted a fundamental contradiction between these Islamic standards, influenced by contemporary jurisprudential trends, and the texts of *Al-Majjahllah* Code, which were influenced by traditional Hanafi jurisprudential trends. This discrepancy was evident in the inconsistency of these contracts with the standards issued by relevant Islamic institutions and the provisions of *Al-Majjahllah*.

The *Ijārah Muntahiyah bi-t-tamlīk* transactions involve a bank's promise to transfer ownership to the lessee if the latter pays all the agreed rent installments. The first challenge arises from the fact that this promise is not legally binding according to the texts of *Al-Majjahllah*. Moreover, Palestinian law governing real estate sales mandates the registration of transactions with land registry departments, and this requirement also applies to promises of sale. However, Islamic banks operating in Palestine do not comply with these requirements, rendering the promise legally ineffective.

Furthermore, the study identified a clear contradiction between the conditions of the *Ijārah Muntahiyah bi-t-tamlīk* contracts issued by Islamic banks in Palestine and the validity conditions set forth by AAOIFI standards. These standards require the contracting parties to uphold the provisions of the lease contract throughout the lease period. Banks are obligated to ensure the property is fit for the intended purpose, cover essential maintenance expenses regularly, pay all government agency fees related to the property, and assume the risks associated with the property. However, the standard contracts deviate from these provisions and transfer all these obligations to the tenant. Additionally, they include excessive penalty clauses that exceed the actual value of the damage suffered by the bank due to the tenant's breach. This aspect contradicts AAOIFI standards and the general legal principles in Palestine.

Considering these findings, the research recommends enacting the Draft of Civil Code to align with contemporary jurisprudential trends of Islamic Fiqh. This Code should state that the contract made by one or both parties to enter a particular contract later will only carry legal weight if all the necessary details of the envisioned contract and the specified timeframe for its completion are clearly outlined. Also, The PMA is recommended to direct Islamic Banks to register the *Ijārah Muntahiyah bi-t-tamlīk* agreements before the land registry departments. Also, the PMA should review the standard contracts issued by Islamic banks to ensure their compliance with contemporary Islamic standards. Banks should bear all substantive obligations in proportion to the content of the lease contract.

## Funding statement

Not applicable.

## Author contribution statement

Mahmoud Fayyad: Conceived and designed the experiments; Performed the experiments; Analyzed and interpreted the data; Contributed reagents, materials, analysis tools or data; Wrote the paper.

## Data availability statement

No data was used for the research described in the article.

## Declaration of competing interest

The authors declare that they have no known competing financial interests or personal relationships that could have appeared to influence the work reported in this paper.

## Footnotes

1 Judgment of the Court of Cassation held in Ramallah in the civil case No. 10 of 2015 issued on 18-1-2018, Judgment of the Court of Cassation held in Ramallah in the civil case No. 348 of 2015 dated 24-12-2017, Judgment of the Court of Cassation held in Ramallah in the civil case No. 1070 of 2015 dated 8-10-2017, Judgment of the Court of Cassation held in Ramallah in the civil case No. 282 of 2014 dated 21-11-2016, Judgment of the Court of Cassation held in Ramallah in the civil case No. 5 of 2015 dated 4-5-2015, Judgment of the Court of Cassation held in Ramallah in the Case No. 184 of 2004 dated 20-2-2005.

2 Judgment of the Court of Cassation held in Ramallah in Case No. 114 of 2007 dated 24-6-2008, Judgment of the Court of Appeal held in Ramallah in Case No. 314 of 2009 dated 8-7-2010, Judgment of the Court of Appeal held in Ramallah in the Case No. 70 of 2008 dated 18-4-2010, Judgment of the Court of Appeal held in Ramallah in the Case No. 251 of 1999 dated 30-10-2000, Judgment of the Court of Appeal held in Ramallah in the Case No. 482 of 2009 dated 9-9-2009.

3 Judgment of the Court of Cassation held in Ramallah in Case No. 248 of 2011 dated 31-3-2013, Judgment of the Court of Cassation held in Ramallah in Case No. 20 of 2009 dated 13-5-2009, Judgment of the Court of Cassation held in Ramallah in Case No. 46 of 2004 dated 5-5-2004.

4 Judgment of the Court of Cassation held in Ramallah in Case No. 22 of 2004 dated 2-5-2004, Judgment of the Court of Cassation held in Ramallah in Case No. 839 of 2015 dated 8-10-2015, Judgment of the Court of Appeal held in Ramallah in Case No. 705 of 1997 dated 14-9-1999, Judgment of the Court of Appeal held in Ramallah in Case No. 382 of 2009 dated 9-3-2011.
